# Integrative analysis illustrates the role of PCDH7 in lung cancer development, cisplatin resistance, and immunotherapy resistance: an underlying target

**DOI:** 10.3389/fphar.2023.1217213

**Published:** 2023-07-19

**Authors:** Huakang Li, Haoran Xu, Hong Guo, Kangming Du, Diang Chen

**Affiliations:** Hospital of Chengdu University of Traditional Chinese Medicine, Chengdu, Sichuan, China

**Keywords:** lung cancer, cisplatin resistance, PCDH7, immunotherapy, target

## Abstract

**Background:** Cisplatin resistance is a common clinical problem in lung cancer. However, the underlying mechanisms have not yet been fully elucidated, highlighting the importance of searching for biological targets.

**Methods:** Bioinformatics analysis is completed through downloaded public data (GSE21656, GSE108214, and TCGA) and specific R packages. The evaluation of cell proliferation ability is completed through CCK8 assay, colony formation, and EdU assay. The evaluation of cell invasion and migration ability is completed through transwell and wound-healing assays. In addition, we evaluated cell cisplatin sensitivity by calculating IC_50_.

**Results:** Here, we found that PCDH7 may be involved in cisplatin resistance in lung cancer through public database analysis (GSE21656 and GSE108214). Then, a series of *in vitro* experiments was performed, which verified the cancer-promoting role of PCDH7 in NSCLC. Moreover, the results of IC_50_ detection showed that PCDH7 might be associated with cisplatin resistance of NSCLC. Next, we investigated the single-cell pattern, biological function, and immune analysis of PCDH7. Importantly, we noticed PCDH7 may regulate epithelial–mesenchymal transition activity, and the local infiltration of CD8^+^ T and activated NK cells. Furthermore, we noticed that patients with high PCDH7 expression might be more sensitive to bortezomib, docetaxel, and gemcitabine, and resistant to immunotherapy. Finally, a prognosis model based on three PCDH7-derived genes (*GPX8*, *BCAR3*, and *TNS4*) was constructed through a machine learning algorithm, which has good prediction ability on NSCLC patients’ survival.

**Conclusion:** Our research has improved the regulatory framework for cisplatin resistance in NSCLC and can provide direction for subsequent related research, especially regarding PCDH7.

## Introduction

Lung cancer is a malignant tumor originating from the lung epithelium and is widely distributed worldwide (Nasim et al., 2019). The onset of lung cancer is extremely complex and is the result of a combination of multiple factors (Mao et al., 2016). From a pathological perspective, lung cancer can be divided into different subtypes, with non-small-cell lung cancer (NSCLC) being the most prominent (Herbst et al., 2018). Surgical intervention remains the first choice for lung cancer, but with the progress of related surgeries, the 5-year survival rate of lung cancer remains poor (Pallis and Syrigos, 2013). The advent of tyrosine kinase inhibitors (TKIs) targeting epidermal growth factor receptor (EGFR) mutations and anaplastic lymphoma kinase (ALK) rearrangements has considerably improved patient survival and the quality of life. Moreover, therapies targeting other genomic alterations, such as ROS1 rearrangements and BRAF, MET, and RET mutations, have emerged (Goldstraw et al., 2016). However, despite these advances, challenges persist. Resistance to first-line TKIs commonly develops, leading to disease progression. Novel strategies like combination therapies and next-generation TKIs are being explored to overcome resistance (Camidge et al., 2012). On one hand, the early symptoms of lung cancer are relatively hidden, and some patients have already lost the opportunity for surgery at the initial diagnosis (Nooreldeen and Bach, 2021). On the other hand, lung cancer has a unique biological specificity, which makes finding specific targets from the perspective of molecular biology helpful for clinical transformation.

Cisplatin is a first-line drug for the treatment of many solid tumors, and it is a heavy metal complex that can inhibit the process of DNA replication (Dasari and Tchounwou, 2014; Ghosh, 2019). Cisplatin combined with specific chemotherapy drugs has achieved a certain efficacy in lung cancer, but it is still limited by multiple adverse reactions and acquired drug resistance (Kryczka et al., 2021). Based on this finding, some researchers have begun to explore the molecular biological mechanisms that affect cisplatin resistance (Galluzzi et al., 2012). Lin et al. noticed that autophagy is involved in cisplatin resistance in pharyngeal squamous cell carcinoma, and this process is induced by RAB3B in extrachromosomal circular DNA (Lin et al., 2022). In addition, researchers found that CAMK2G phosphorylated ITPKB by ROS in ovarian cancer, leading to resistance to cisplatin (Li et al., 2022). Ni et al. discovered that the combination of shikonin and cisplatin promotes ferroptosis by upregulating HMOX1, further overcoming cisplatin resistance in ovarian cancer (Ni et al., 2023). In lung cancer, Xiao et al. demonstrated that RAP1 can activate NF-κB signaling and mediate cisplatin resistance of NSCLC (Xiao et al., 2017). Interestingly, Ray et al. revealed that nicotine may affect cisplatin resistance in lung cancer, indicating the importance of lifestyle interventions for patients (Ray et al., 2022). Wu et al. found that the exosome miR-193a can lead to cisplatin resistance of NSCLC by targeting LRRC1 (Wu et al., 2020). Consequently, exploring the factors and potential targets that affect cisplatin resistance from a molecular biology perspective is of great significance.

As sequencing technology developed, massive second-generation sequencing data have been generated and are publicly available, providing great convenience for researchers (Ren et al., 2020; Zhang et al., 2021a; Zhang et al., 2021b; Zhang et al., 2022). Here, we found that PCDH7 may be involved in cisplatin resistance in lung cancer through public database analysis (GSE21656 and GSE108214). Then, a series of *in vitro* experiments was performed, which verified the cancer-promoting role of PCDH7 in NSCLC. Moreover, the results of IC_50_ detection showed that PCDH7 might be associated with cisplatin resistance of NSCLC. Next, we investigated the single-cell pattern, biological function, and immune analysis of PCDH7. Moreover, we noticed that high PCDH7 expression might be more sensitive to bortezomib, docetaxel, and gemcitabine. Finally, a prognosis model based on three PCDH7-derived genes was constructed (*GPX8*, *BCAR3*, and *TNS4*), which has a good prediction ability on NSCLC patients’ survival.

## Methods

### Collection of public data

For The Cancer Genome Atlas (TCGA) database, we downloaded the original transcriptome data from TCGA–GDC (TCGA–LUAD and –LUSC projects; STAR-Counts form). Before conducting the analysis, we merged and organized the downloaded raw transcriptional data into an expression matrix. A human genome reference document is used for ENSG number annotation. Meanwhile, we performed mean taking on duplicate genes, and genes with an average expression of less than 0.05 were deleted. The clinical formation was also obtained from the TCGA–GDC (bcr-xml form). For the Gene Expression Omnibus database, the data from GSE21656 and GSE108214 were selected, which provided the next-sequence data from cisplatin-resistant and wild-type lung cancer cells (Sun et al., 2012; Sarin et al., 2018). The probe annotation of GSE21656 was conducted using GPL6244, and GSE108214, using GPL17077. The baseline information on HNSCC patients from TCGA database is shown in Table 1.

**TABLE 1:** Baseline information on the enrolled patients.

Clinical feature		Number of patients	Percentage (%)
Age	<=65	431	42.0
	>65	567	55.3
	Unknown	28	2.7
Gender	Female	411	40.1
	Male	615	59.9
Stage	Stage I	524	51.1
	Stage II	287	28.0
	Stage III	170	16.6
	Stage IV	33	3.2
	Unknown	12	1.2
T-stage	T1	286	27.9
	T2	576	56.1
	T3	118	11.5
	T4	43	41.9
	Unknown	3	0.3
M-stage	M0	767	74.8
	M1	32	3.1
	Unknown	227	22.1
N-stage	N0	655	63.8
	N1	231	22.5
	N2	115	11.2
	N3	7	0.7
	Unknown	18	1.8

### Bioinformatics analysis

The limma package was applied for differentially expressed gene (DEG) analysis with specific thresholds (Ritchie et al., 2015). By integrating patient expression profiles and prognostic data, univariate Cox regression analysis was utilized to identify the genes remarkably correlated with patient survival with a *p* < 0.05. Pathway enrichment was explored using the gene set enrichment analysis (GSEA) algorithm (Subramanian et al., 2005). The Gene Ontology (GO), Hallmark, and Kyoto Encyclopedia of Genes and Genomes (KEGG) gene sets were selected as the reference set. The immune microenvironment was quantified using the CIBERSORT algorithm based on the input expression matrix (Chen et al., 2018). Quantification of immune function was completed using the single-sample GSEA (ssGSEA) algorithm (Hänzelmann et al., 2013). Quantification of the stromal score, immune score, and estimate score was conducted using the ESTIMATE package (Yu et al., 2021a). The response of patients to immunotherapy was quantified using the Tumor Immune Dysfunction and Exclusion (TIDE) algorithm (Fu et al., 2020). The TIDE algorithm gives each patient a TIDE score. Lung cancer patients with TIDE scores more than zero were defined as responders to immunotherapy, and those with scores less than zero were the opposite. Quantification of patients on target drugs was realized using the Genomics of Drug Sensitivity in Cancer (GDSC) database (Yang et al., 2013). The machine learning algorithm LASSO regression was utilized to reduce the data dimension (McEligot et al., 2020). The prognosis prediction model was identified using the multivariate Cox regression analysis. For a better clinical application, a nomogram that merges the risk score and clinical features was constructed. The prediction value of one certain continuous variable to survival was performed using the receiver operating characteristic (ROC) curve.

### Single-cell analysis

Single-cell analysis is directly completed through an online interactive website—the TISCH project (Sun et al., 2021). GSE148071 was conducted by Wu et al. (2021). In this project, they collected 42 NSCLC samples and characterized the entire tumor ecosystem through single-cell RNA sequencing.

### Immunohistochemistry (IHC) and subcellular localization

The IHC image of PCDH7 in lung cancer and para-carcinoma tissue was directly downloaded from The Human Protein Atlas (HPA) project (Colwill and Gräslund, 2011). Subcellular localization of PCDH7 in the HPA project was obtained in the HeLa cells.

### Cell culture

The used BEAS-2B, A549, H1299, H522, H460 cells, and A549-Res cell lines (A549 cell line that is resistant to cisplatin) were routinely stored in laboratories. The A549-Res (resistant to cisplatin) cell line was purchased from Shanghai MEIXUAN Biological Science and Technology Co, Ltd. All cells are cultured under normal conditions. The cell culture medium used is 1640-RPMI. According to cell growth, subculture was conducted every 3–4 days.

### Quantitative real-time (qRT) PCR

First, total RNA was extracted using the TRIzol reagent and then transcribed into cDNA for further analysis (Pan et al., 2020). The PCR system is a 20-μL system, and PCR detection is performed using SYBR Green. The primer sequence is as follows: PCDH7: F, 5′-GAC​TCT​GGG​CGT​CTC​TGA​AG-3’; R, 5′-CTC​AAC​TCC​GAC​TCT​GCT​CA; GAPDH: F, 5′-CTG​GGC​TAC​ACT​GAG​CAC​C-3’; R, 5′-AAG​TGG​TCG​TTG​AGG​GCA​ATG-3’.

### Cell transfection

The transfection of control and PCDH7 knockdown plasmids was conducted with Lipofectamine 2000 based on standard procedures (Pan et al., 2020). The target sequence is as follows: sh#1: 5′-GGA​GGC​TTC​TAA​GCC​AAA​T-3′, sh#2: 5′-GGA​CCA​TTT​ACT​CCA​CAA​T-3′, sh#3: 5′-CCA​CCG​TGG​TCC​TTA​ACA​T-3’.

### Cell proliferation

The proliferation ability of A549 and H1299 cells was evaluated using the CCK8, colony formation, and EdU assays, according to standard procedures (Pan et al., 2020).

### Transwell and wound-healing assays

The invasion and migration ability of A549 and H1299 cells were evaluated using the transwell and wound-healing assays, according to standard procedures (Pan et al., 2020).

### IC_50_ detection of cisplatin

The detection of cisplatin was performed according to the procedures of Heinze et al. (2021).

### *In vivo* experiments

For tumor inoculation, each nude mouse was subcutaneously injected with 10 × 10^5^ cells. The mice were then monitored for 4 weeks for tumor development. At the end of the experiment, the mice were euthanized, and tumors were excised, weighed, and photographed.

### Data statistics

For all the analyses based on public data, R software version 4.0.4 was used. Moreover, SPSS and GraphPad Prism 8 software were also used for data statistics of the experimental data. Generally, the comparison with a *p*-value <0.05 was regarded as significant. Different testing methods were chosen based on statistical requirements for variables that meet different data distributions.

## Results

**FIGURE 1:**
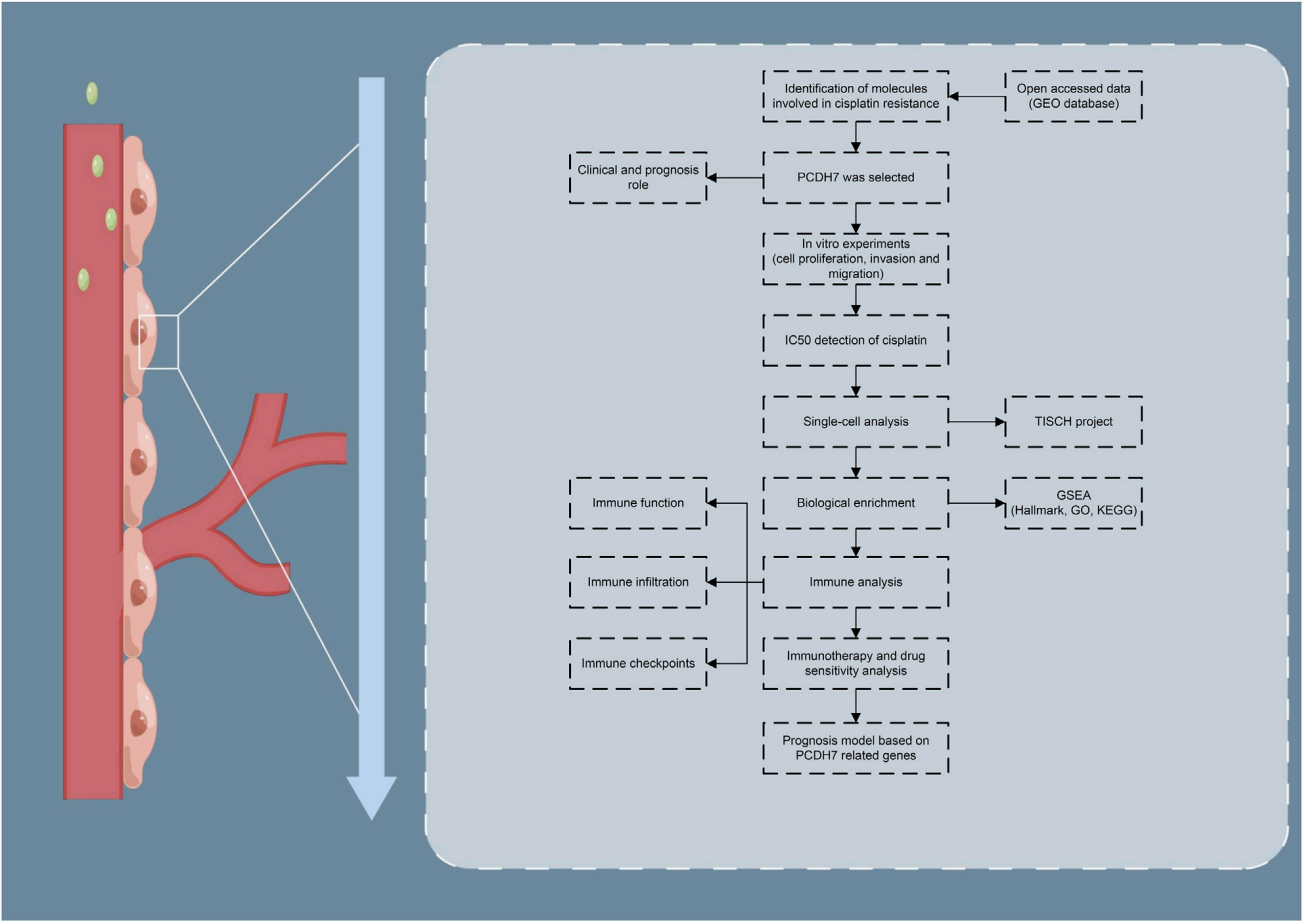
Flow chart of the whole study.

The brief process of this study is shown in Figure 1. Here, we found that PCDH7 may be involved in cisplatin resistance in lung cancer through public database analysis (GSE21656 and GSE108214). Then, a series of *in vitro* experiments was performed, which verified the cancer-promoting role of PCDH7 in NSCLC. Moreover, the results of IC_50_ detection showed that PCDH7 might be associated with cisplatin resistance of NSCLC. Next, we investigated the single-cell pattern, biological function, and immune analysis of PCDH7. Moreover, we noticed that patients with high PCDH7 expression might be more sensitive to bortezomib, docetaxel, and gemcitabine but resistant to immunotherapy. Finally, a prognosis model based on three PCDH7-derived genes was constructed (*GPX8*, *BCAR3*, and *TNS4*), which has a good prediction ability on NSCLC patients’ survival.

### Identification of the genes involved in cisplatin resistance in lung cancer cells

**FIGURE 2:**
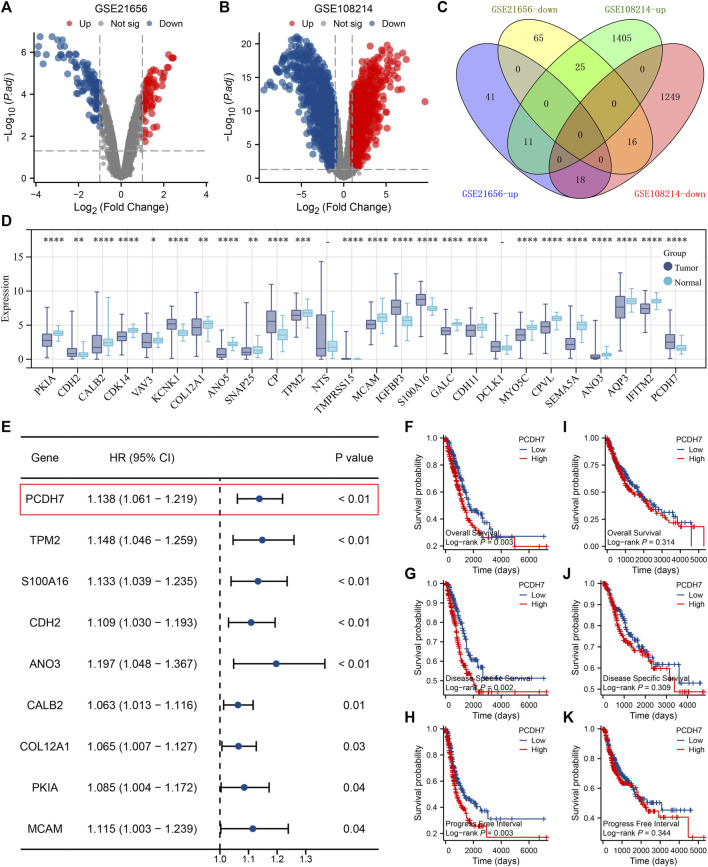
Identification of PCDH7 through bioinformatics analysis. Notes: **(A)** DEG analysis between cisplatin-resistant and wild-type cells in GSE21656; **(B)** DEG analysis between cisplatin-resistant and wild-type cells in GSE108214. **(C)** Intersection analysis of the DEGs identified from GSE21656 and GSE108214. **(D)** Expression patterns of intersected genes in NSCLC and para-carcinoma tissues. **(E)** Univariate Cox regression analysis was performed to identify the prognosis-related genes. **(F)** Overall survival difference in patients with high and low PCDH7 expression (TCGA–LUAD). **(G)** Disease-free survival difference in patients with high and low PCDH7 expression levels (TCGA–LUAD). **(H)** Progression-free survival difference in patients with high and low PCDH7 expression levels (TCGA–LUAD). **(I)** Overall survival difference in patients with high and low PCDH7 expression (TCGA–LUSC). **(J)** Disease-free survival difference in patients with high and low PCDH7 expression (TCGA–LUSC). **(K)** Progression-free survival difference in patients with high and low PCDH7 expression (TCGA–LUSC).

**FIGURE 3:**
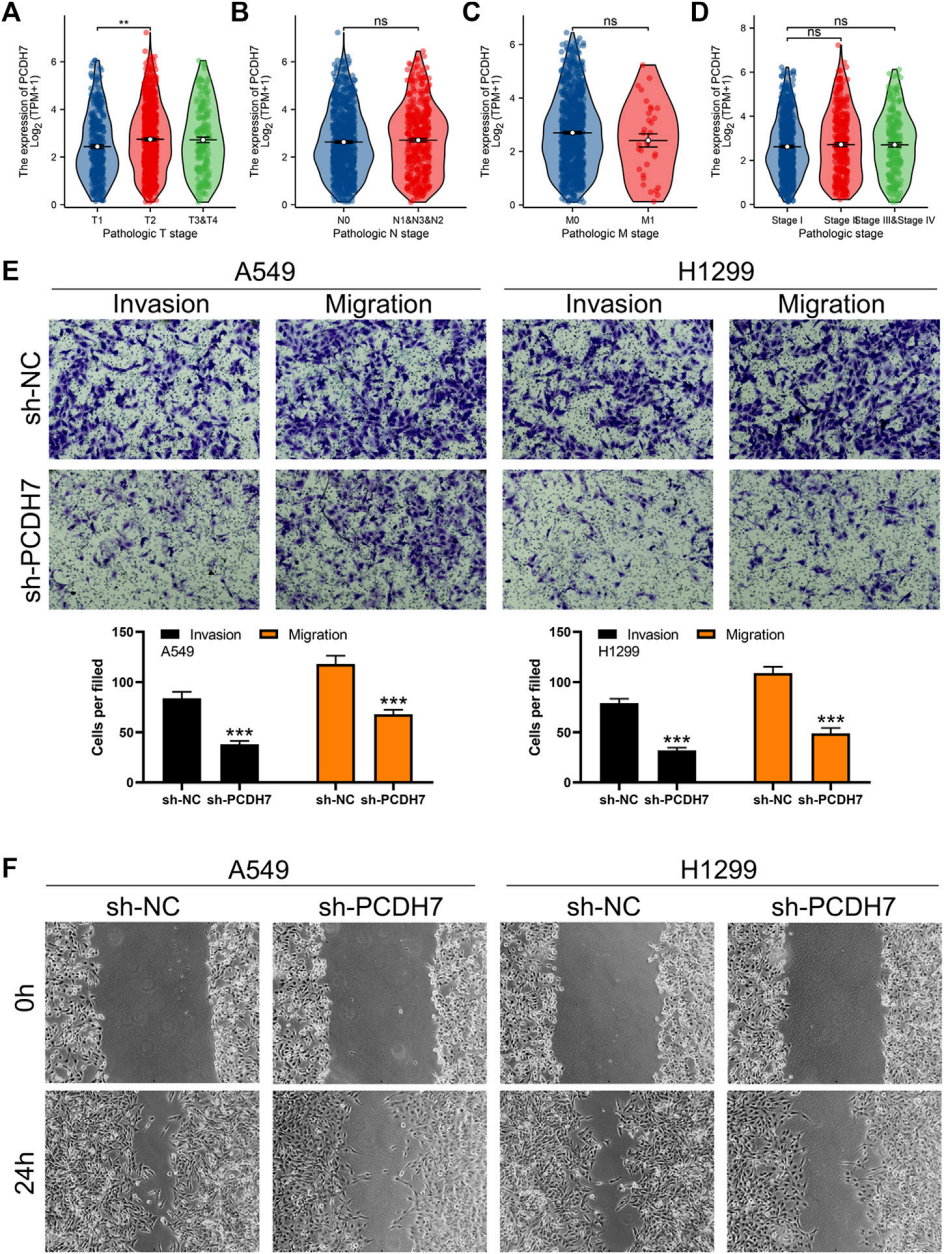
PCDH7 promotes the invasion and migration ability of NSCLC. Notes: **(A–D)** expression level of PCDH7 in patients with different clinical features. **(E)** Transwell assay was performed in PCDH7-knockdown and control cells. **(F)** Wound-healing assay was performed in PCDH7-knockdown and control cells.

Through careful search, we found two datasets from the GEO database. The GSE21656 provided the transcriptional profile derived from cisplatin-resistant and wild-type H460 lung cancer cells. GSE108214 provided the transcriptional profile derived from cisplatin-resistant and wild-type A549 lung cancer cells. The data preprocessing process is shown in Supplementary Figures S1A, B. The DEG analysis identified 70 upregulated and 106 downregulated genes in cisplatin-resistant cells of the GSE21656 cohort (Figure 2A, H460). For the GSE108214 cohort, 1,430 upregulated and 1,280 downregulated genes were identified in cisplatin-resistant A549 cells (Figure 2B). Through intersection processing, we found that 16 genes showed consistent downregulation in drug-resistant cells in the GSE21656 and GSE108214 cohorts: *NTS*, *TMPRSS15*, *TMEM27*, *MCAM*, *IGFBP3*, *S100A16*, *GALC*, *CDH11*, *DCLK1*, *MYO5C*, *CPVL*, *SEMA5A*, *ANO3*, *AQP3*, *IFITM2*, and *PCDH7*; in total, 11 genes showed consistent upregulation in drug-resistant cells in the GSE21656 and GSE108214 cohorts: *PKIA*, *CDH2*, *CALB2*, *CDK14*, *VAV3*, *KCNK1*, *COL12A1*, *ANO5*, *SNAP25*, *CP*, and *TPM2* (Figure 2C). Then, we compared the expression level of these common genes in NSCLC and para-carcinoma tissue. We noticed most of these genes had a significant difference in the expression level between tumor and normal tissues, revealing their underlying role in cancer development (Figure 2D). The results of univariate Cox regression showed that the genes *PCDH7*, *TPM2*, *S100A16*, *CDH2*, *ANO3*, *CALB2*, *COL12A1*, *PKIA*, and *MCAM* were remarkably correlated with NSCLC patient survival (Figure 2E, *PCDH7*: HR = 1.138; *TPM2*: HR = 1.148; *S100A16*: HR = 1.133; *CDH2*: HR = 1.109; *ANO3*: HR = 1.197; *CALB2*: HR = 1.063; *COL12A1*: HR = 1.065; *PKIA*, HR = 1.085; *MCAM*, HR = 1.115). Among these genes, *PCDH7* has the most significant *p*-value and was consequently selected for the following analysis. In the TCGA–LUAD cohort (lung adenocarcinoma), patients with high expression of *PCDH7* appear to have a poor prognosis (Figures 2F–H, overall survival, *p* = 0.003; disease-free survival, *p* = 0.002; progression-free survival, *p* = 0.003). However, this effect does not seem significant in the TCGA–LUSC cohort (lung squamous cell carcinoma) (Figures 2I, J). Furthermore, an association between PCDH7 and worse clinical features was found in a clinical correlation analysis (Figures 3A–D).

### PCDH7 enhances the cell malignant phenotypes and cisplatin resistance of NSCLC cells

**FIGURE 4:**
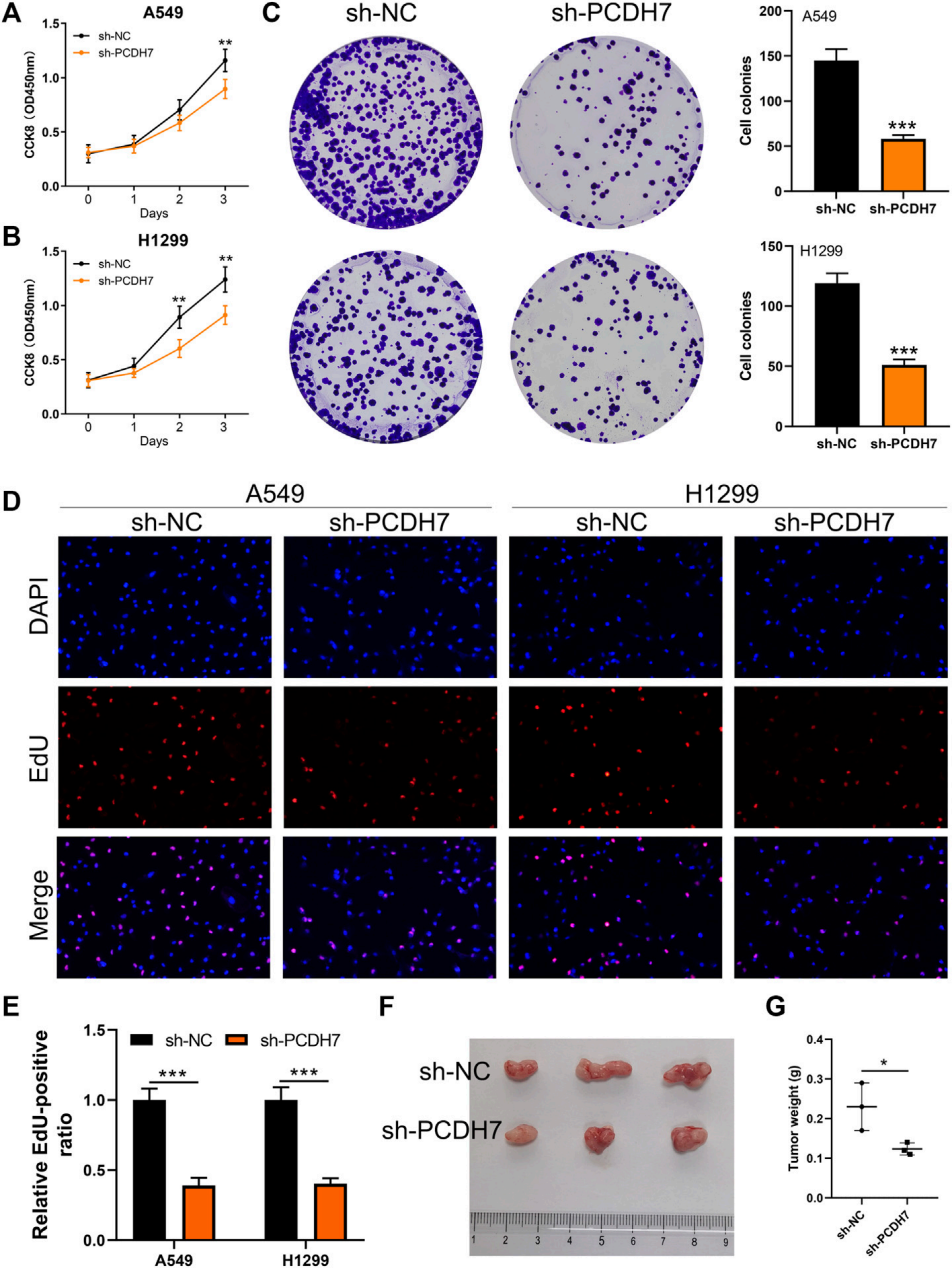
PCDH7 promotes the proliferation ability of NSCLC. Notes: **(A, B)** CCK8 assay was performed in PCDH7-knockdown and control cells. **(C)** Colony formation assay was performed in PCDH7-knockdown and control cells. **(D, E)** EdU assay was performed in PCDH7-knockdown and control cells. **(F, G)** Tumor formation experiment in nude mice injected by control and PCDH7-knockdown cells.

**FIGURE 5:**
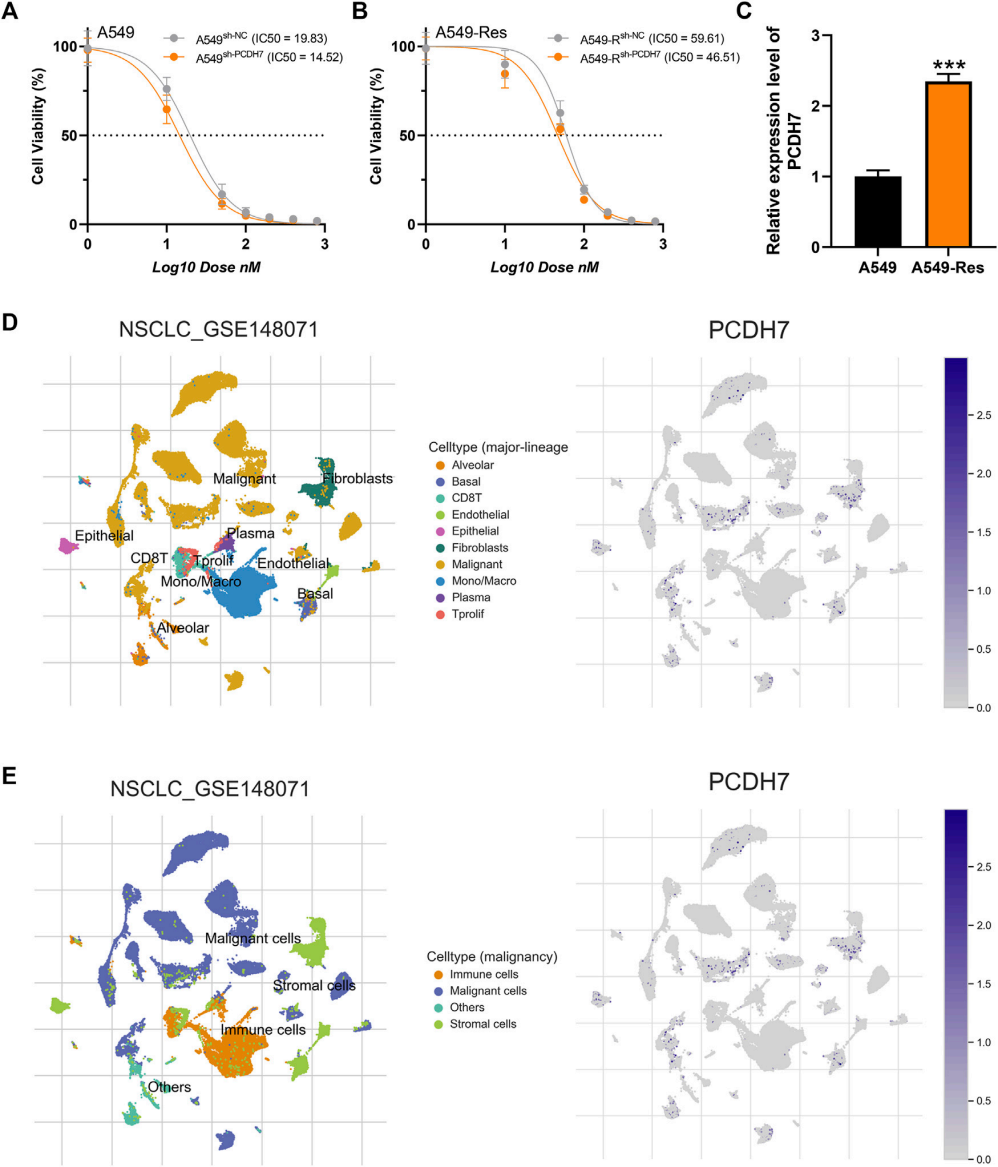
PCDH7 affects cisplatin resistance in NSCLC. Notes: **(A, B)** The IC_50_ detection of cisplatin in PCDH7-knockdown and control cells. **(C)** Expression level of PCDH7 in A549- and A549-resistant cells. **(D, E)** Single-cell expression pattern in NSCLC tissue.

Subsequently, we tried to identify the biological role of PCDH7 in NSCLC. The results of qRT-PCR indicated that PCDH7 is overexpressed in lung cancer cells compared to normal lung cells (Supplementary Figure S2A). Moreover, the IHC image from the HPA database indicated a higher PCDH7 protein level in NSCLC than the control tissue (Supplementary Figures S3A, B). The inhibition efficiency of three sh-PCDH7 cells was quantified using qRT-PCR (Supplementary Figures S2B, C). In both A549 and H1299 cells, sh-PCDH7#2 showed the best performance and was consequently selected for further experiments. The transwell assay indicated that knockdown of PCDH7 could hamper the invasion and migration ability of NSCLC cells (Figure 3E). The result of the wound-healing assay obtained the same conclusion (Figure 3F). For cell proliferation, the results of the CCK8 assay indicated that inhibition of PCDH7 could reduce the cell proliferation ability of NSCLC cells (Figures 4A, B). In addition, the number and size of cell colonies in cells with PCDH7 knockdown were smaller than those in control cells (Figure 4C). The EdU assay indicated that inhibition of PCDH7 could remarkably inhibit the DNA replication capability of NSCLC cells (Figures 4D, E). *In vivo* experiments showed that PCDH7 knockdown cells formed a lighter tumor than control cells (Figures 4F, G). Then, we detected the IC_50_ concentration of cisplatin in sh-PCDH7 and control cells. The result indicated that cells with sh-PCDH7 had a lower IC_50_ than control cells, indicating that PCDH7 is associated with cisplatin resistance (Figures 5A, B). Moreover, A549 cells with cisplatin resistance had a higher PCDH7 level (Figure 5C).

### Expression pattern and biological function of PCDH7 in NSCLC

**FIGURE 6:**
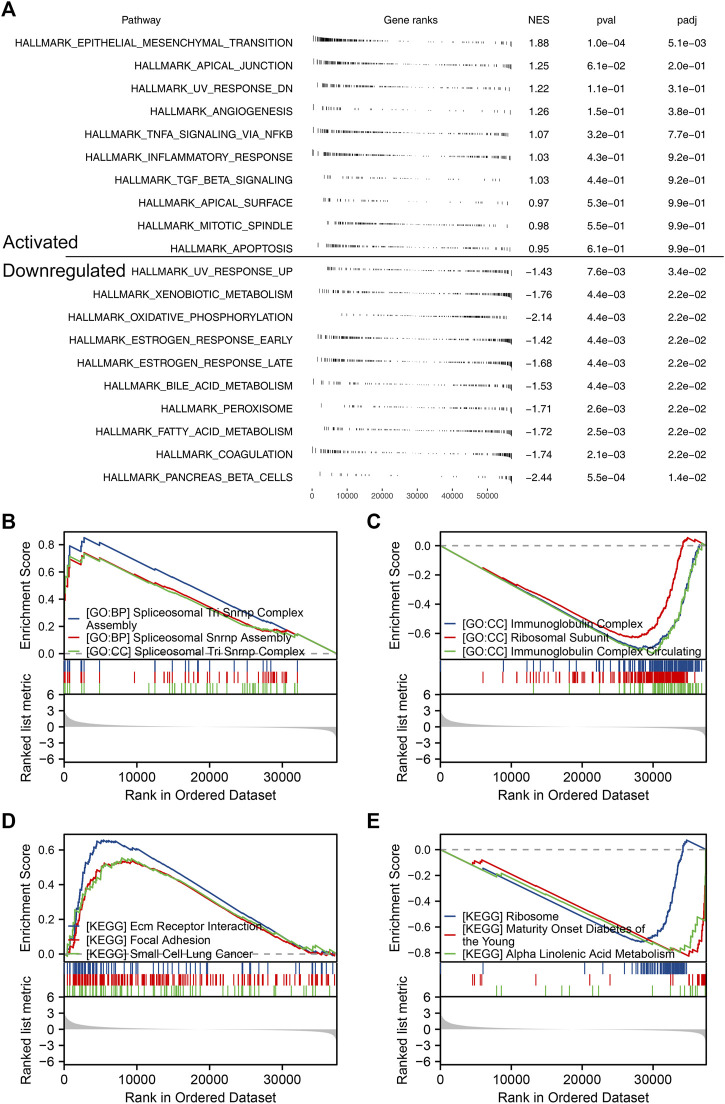
Biological enrichment of PCDH7 in NSCLC. Notes: **(A)** GSEA analysis based on the Hallmark gene set in NSCLC patients with high and low PCDH7 expression. **(B)** Top three upregulated terms of GSEA analysis based on the GO gene set in NSCLC patients with high and low PCDH7 expression. **(C)** Top three downregulated terms of GSEA analysis based on the GO gene set in NSCLC patients with high and low PCDH7 expression. **(D)** Top three upregulated terms of GSEA analysis based on the KEGG gene set in NSCLC patients with high and low PCDH7 expression. **(E)** Top three downregulated terms of GSEA analysis based on the KEGG gene set in NSCLC patients with high and low PCDH7 expression levels.

Based on the public single-cell data from TISCH projects (Wu et al., 2021), we explored the single-cell expression pattern of PCDH7 in NSCLC. The result showed that PCDH7 was mainly expressed in malignant cells, fibroblasts, and CD8^+^ T cells (Figures 5D, E). The GSEA analysis based on the Hallmark set indicated that in the patients with high PCDH7 expression, the top five upregulated terms were epithelial–mesenchymal transition (EMT), apical junction, UV-response, angiogenesis, and TNF-α signaling (Figure 6A). For the GSEA analysis based on the GO set, the top three upregulated terms were all related to the spliceosome-related complex (Figure 6B) and the top three downregulated terms were related to the immunoglobulin complex (Figure 6C). For the GSEA analysis based on the KEGG set, the top three upregulated terms were small-cell lung cancer, focal adhesion, and ECM–receptor interaction, while the top three downregulated terms were ribosome, maturity-onset diabetes of the young, and linolenic acid metabolism (Figures 6D, E). The subcellular localization of PCDH7 in HeLa cells showed that it mainly localized in the plasma membrane (Supplementary Figures S3C, D).

### PCDH7 affects the immune microenvironment and therapy response of NSCLC

**FIGURE 7:**
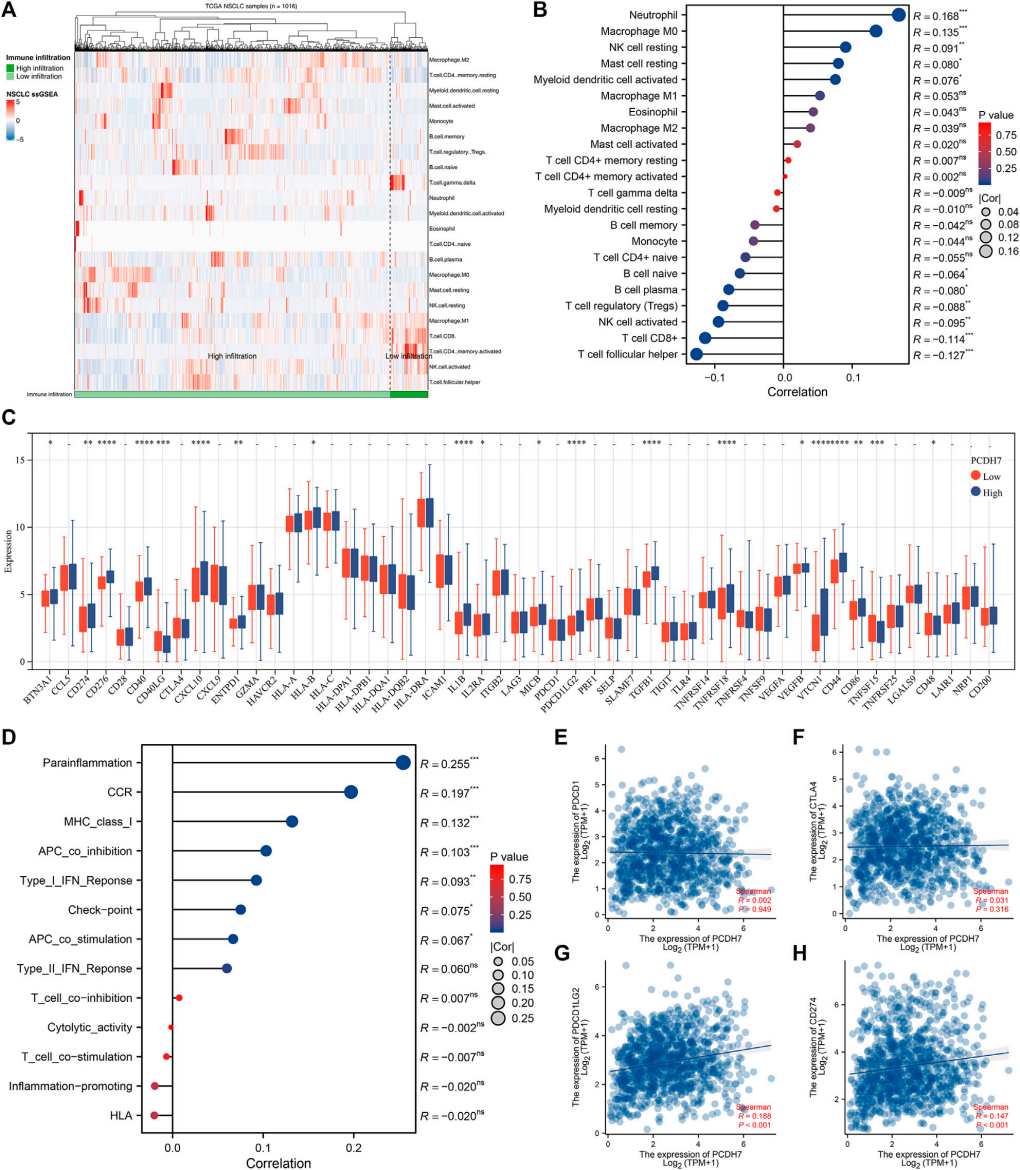
Immune-related analysis of PCDH7 in NSCLC. Notes: **(A)** The CIBERSORT algorithm was used to quantify the immune microenvironment of NSCLC. **(B)** Correlation between PCDH7 and quantified immune cells. **(C)** Expression level of immune checkpoint genes in patients with high and low PCDH7 expression. **(D)** Correlation between PCDH7 and quantified immune function. **(E)** Correlation between PCDH7 and PDCD1. **(F)** Correlation between PCDH7 and CTLA4. **(G)** Correlation between PCDH7 and PDCD1LG2. **(H)** Correlation between PCDH7 and CD274.

**FIGURE 8:**
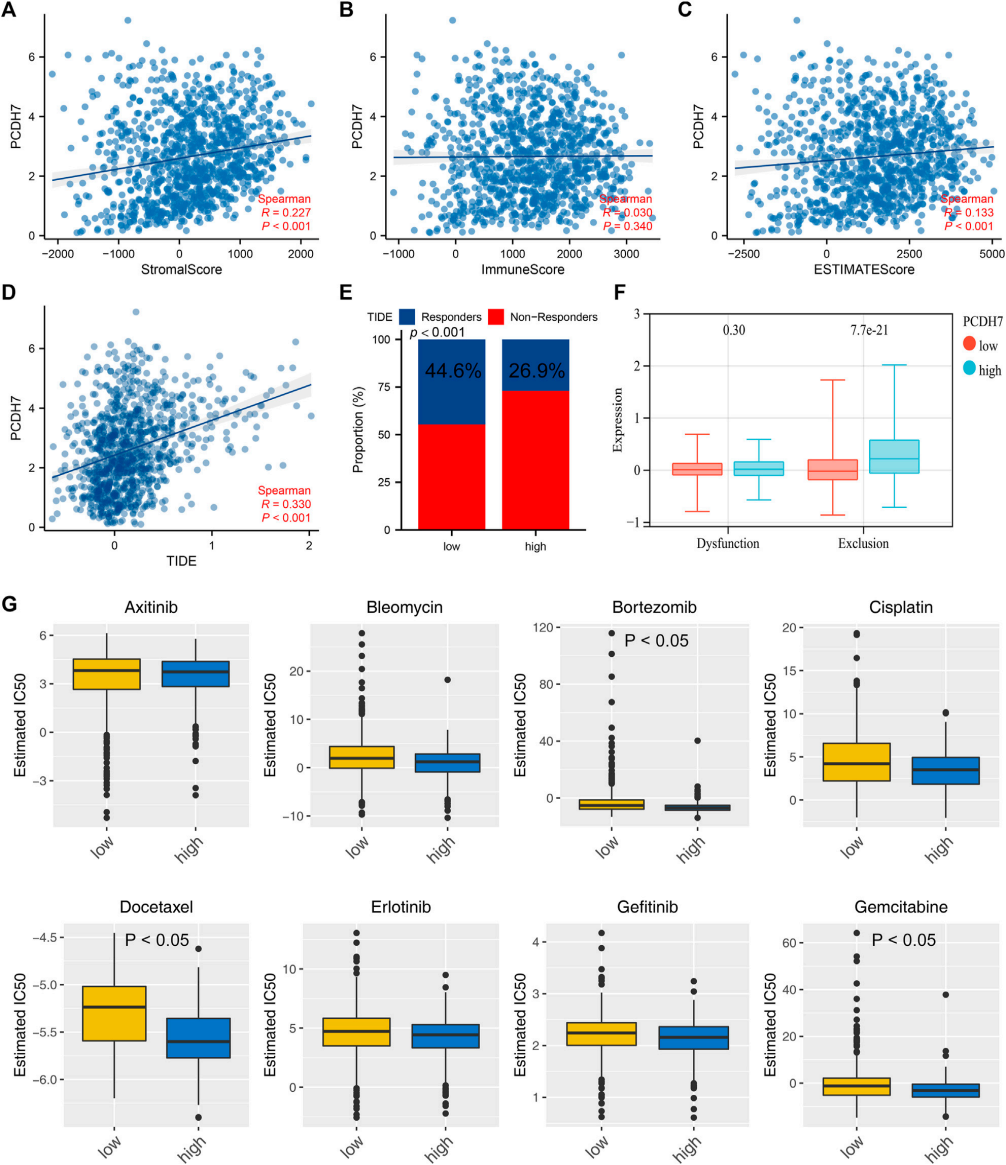
PCDH7 affects the sensitivity of immunotherapy and specific chemotherapy in NSCLC. Notes: **(A)** Correlation between PCDH7 and stromal score quantified by the ESTIMATE package. **(B)** Correlation between PCDH7 and immune score quantified by the ESTIMATE package. **(C)** Correlation between PCDH7 and ESTIMATE score quantified by the ESTIMATE package. **(D)** Correlation between PCDH7 and the TIDE score. **(E)** Percentage of immunotherapy responders and non-responders in patients with high and low PCDH7 expression. **(F)** Immune dysfunction and exclusion level in patients with high and low PCDH7 expression. **(G)** Drug sensitivity analysis of patients with high and low PCDH7 expression.

The heatmap of the level of immune cells quantified by the CIBERSORT algorithm is shown in Figure 7A. Correlation analysis indicated that PCDH7 was positively correlated with resting NK cells, M0 macrophages, and neutrophils yet negatively correlated with CD8^+^ T cells and activated NK cells (Figure 7B). We also noticed a significant difference in several immune-related genes in patients with high and low PCDH7 expression levels (Figure 7C). For the immune function terms quantified by the ssGSEA algorithm, PCDH7 was positively correlated with para-inflammation, CCR, MHC_class_I, and APC_co_inhibition (Figure 7D). Furthermore, we found that PCDH7 was significantly correlated with key immune checkpoints PDCD1LG2 and CD274 (Figures 7E–H). A positive correlation was found between PCDH7 and stromal score, as well as ESTIMATE scores (Figures 8A–C). Moreover, we found that PCDH7 was positively correlated with the TIDE score (Figure 8D, R = 0.330, *p* < 0.001). Correspondingly, 44.6% of patients with low PCDH7 levels tended to respond to immunotherapy, but this percentage reduced to 26.9% in patients with high PCDH7 expression (Figure 8E). Meanwhile, a higher immune exclusion was found in patients with high PCDH7 expression (Figure 8F). The results of drug sensitivity indicated that patients with high PCDH7 expression might be more sensitive to bortezomib, docetaxel, and gemcitabine (Figure 8G).

### Construction of a prognosis model derived from PCDH7 using machine learning algorithms

**FIGURE 9:**
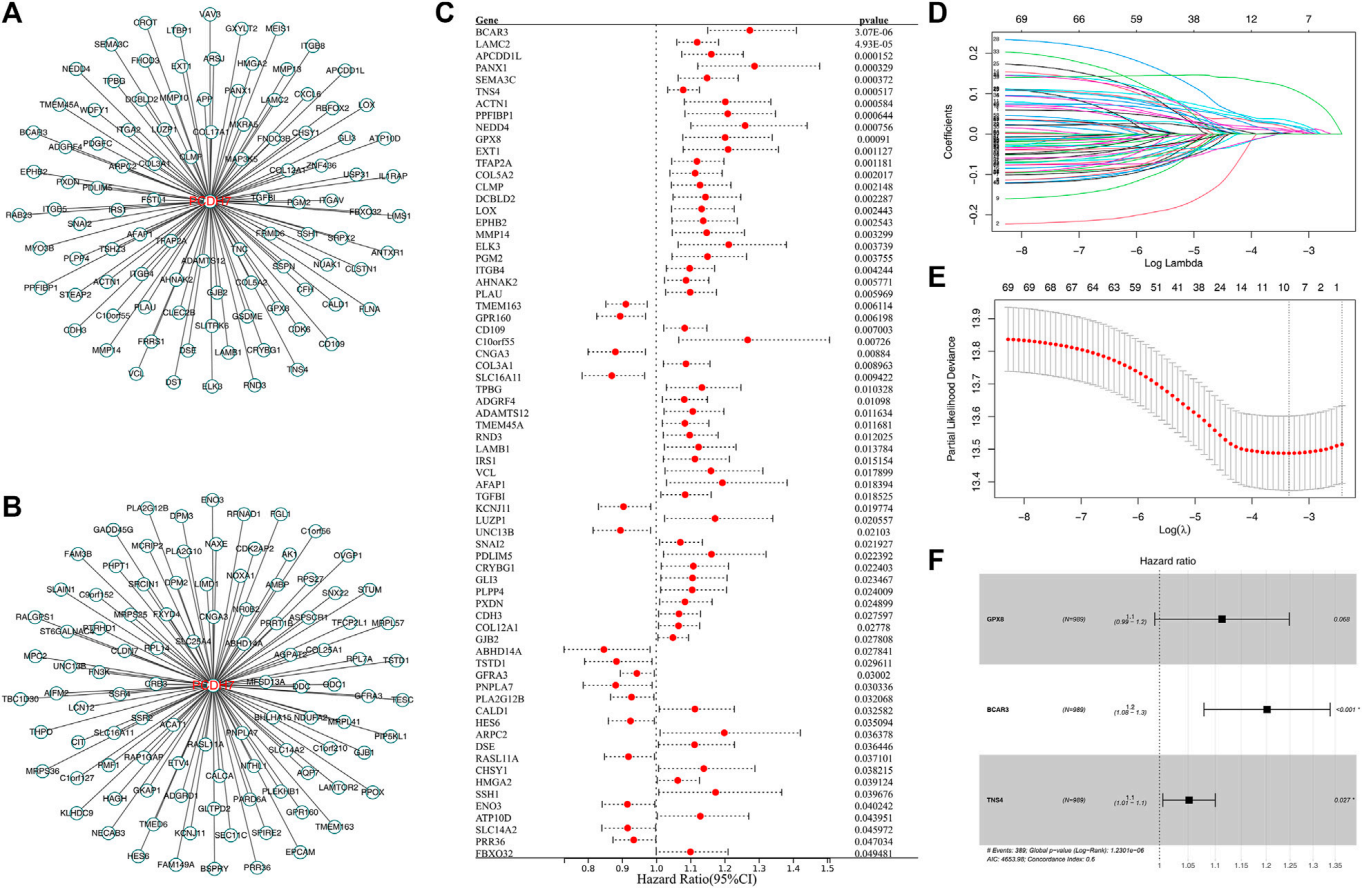
Construction of a prognosis model based on PCDH7-derived molecules. Notes: **(A)** Top 100 genes positively correlated with PCDH7. **(B)** Top 100 genes negatively correlated with PCDH7. **(C)** Univariate Cox regression analysis of PCDH7-derived molecules. **(D, E)** LASSO regression analysis. **(F)** Multivariate Cox regression analysis was performed to construct the prognosis model.

**FIGURE 10:**
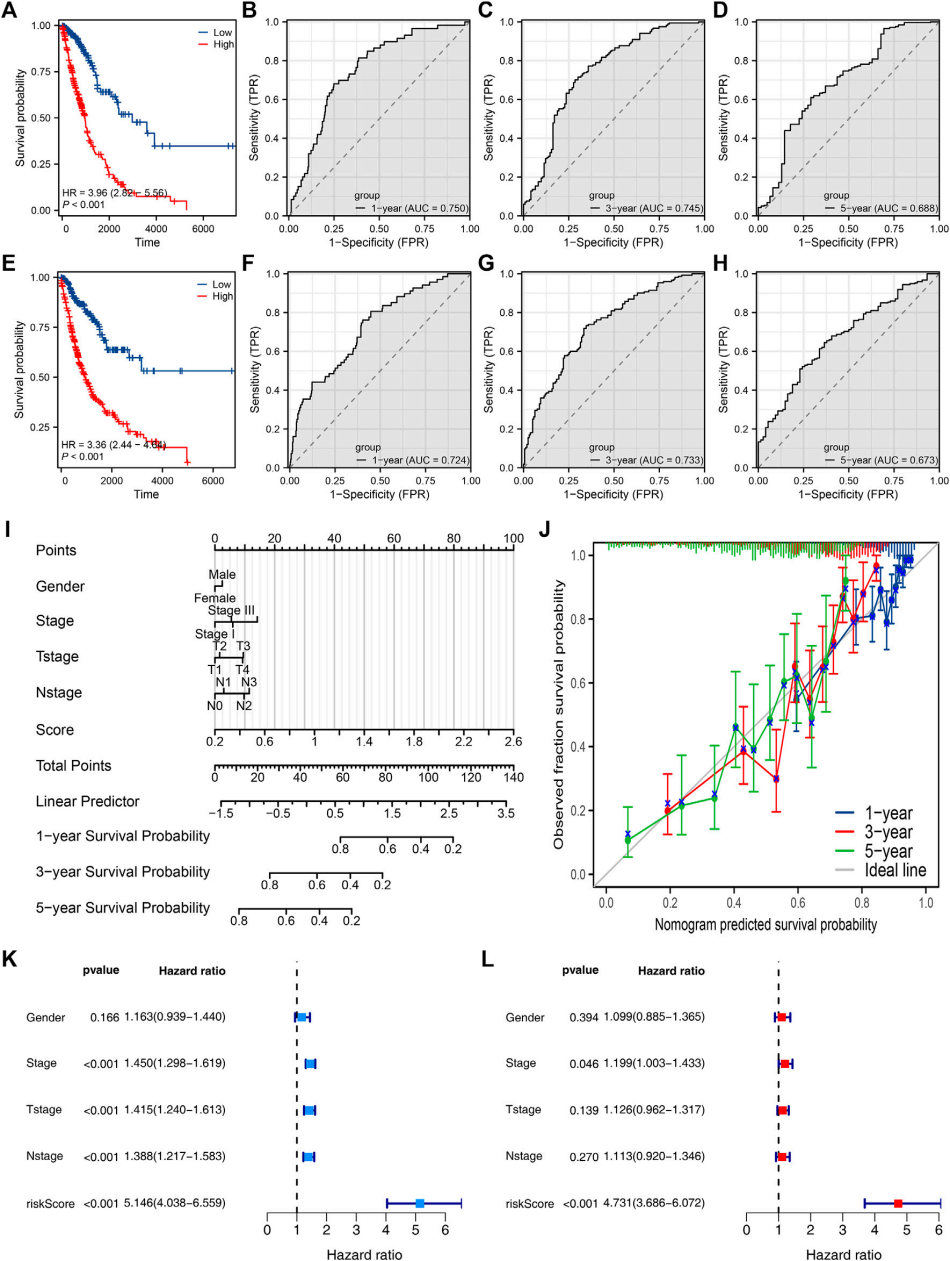
Evaluation of the prognosis model and nomogram plot. Notes: **(A)** KM survival curves of high- and low-risk patients in the training cohort; **(B–D)** ROC curves of 1-, 3-, and 5-year survival in the training cohort; **(E)** KM survival curves of high- and low-risk patients in the validation cohort; **(F–H)** ROC curves of 1-, 3-, and 5-year survival in the validation cohort. **(I)** Nomogram plot constructed by combining the risk score and clinical features. **(J)** Calibration curves were used to evaluate the fit between the nomogram-predicted survival and the actual survival. **(K, L)** Univariate and multivariate analyses of the prognosis model.

Then, we tried to construct a prognosis model derived from PCDH7-related genes. The top 100 genes positively and negatively correlated with PCDH7 are shown in Figures 9A, B. Then, univariate Cox regression analysis was performed to identify the prognosis-related genes (Figure 9C; Supplementary File S1). LASSO regression analysis was conducted to reduce data dimensions (Figures 9D, E). Then, three PCDH7-related molecules were identified for a prognosis model: Risk score = GPX8 * 0.107 + BCAR3 * 0.184 + TNS4 * 0.05 (Figure 9F). The KM curve in the training cohort demonstrated a shorter survival rate of patients with a high risk score than those with a low risk score (Figure 10A, HR = 3.96, *p* < 0.001). The AUC values of 1-, 3-, and 5-year ROC curves were 0.750, 0.745, and 0.688, respectively, indicating a good prediction ability of our prognosis model (Figures 10B–D). This effect was also found in the validation cohort (Figures 10E–H, HR = 3.36, *p* < 0.001; AUC values of 1-, 3-, and 5-year ROC curves were 0.724, 0.733, and 0.673, respectively). A nomogram plot was established to get a better clinical application ability by integrating the risk score and clinical features (Figure 10I). For the 1-, 3-, and 5-year survival, a satisfactory fit was observed between the survival predicted by the nomogram and the actual survival (Figure 10J). Moreover, we noticed that the risk score is an independent marker for patient prognosis, which increases its potential for clinical applications (Figures 10K, L).

## Discussion

Despite the rapid development of medical management and technology, it is undeniable that lung cancer, especially NSCLC, remains a thorny public health issue (de Sousa and Carvalho, 2018; Bade and Dela Cruz, 2020). Early-stage lung cancer patients can rely on early surgery, but surgical intervention in late-stage patients often has poor results (Hirsch et al., 2017). Early-stage lung cancer can achieve a good prognosis and even clinical cure through multidisciplinary comprehensive treatment. However, many patients already have disease progression at the initial diagnosis, which is an important factor affecting their prognosis (Wang et al., 2019). For unresectable advanced NSCLC patients, platinum-containing dual-drug chemotherapy remains the first-line treatment strategy (Rossi and Di Maio, 2016). Therefore, cisplatin is extremely important in the treatment of advanced lung cancer.

In recent years, NSCLC has made great progress in both immune and targeted therapies, and these advances have also promoted the development of precision therapy (Imyanitov et al., 2021). Subsequently, the development of targeted drugs and their molecular therapeutic mechanisms have received attention. However, due to the limitations of high cost and off-target effects of precise medical treatment for tumors, the combination therapy of traditional chemotherapy is still indispensable in clinical treatment (Planchard et al., 2018). Currently, the issue of chemotherapy drug resistance has become a significant obstacle to the treatment of NSCLC. Cisplatin is the main chemotherapy drug for NSCLC, which can damage tumor DNA, inhibit tumor cell mitosis, and thus disrupt a series of biological functions of DNA (Makovec, 2019). The resistance mechanism of cisplatin is very complex, and its resistance is often related to “drug pump” proteins, molecular detoxification, DNA damage repair, and activation of certain pathways (Amable, 2016). Some previous studies have begun to focus on the mechanism of cisplatin resistance and potential intervention targets (Wang et al., 2021; Kouba et al., 2022; Shi et al., 2022). Here, we found that PCDH7 may be involved in cisplatin resistance in lung cancer through public database analysis (GSE21656 and GSE108214). Then, a series of *in vitro* experiments was performed, which verified the cancer-promoting role of PCDH7 in NSCLC. Moreover, the results of IC_50_ detection showed that PCDH7 might be associated with cisplatin resistance of NSCLC. Next, we investigated the single-cell pattern, biological function, and immune analysis of PCDH7. Moreover, we noticed that patients with high PCDH7 expression might be more sensitive to bortezomib, docetaxel, and gemcitabine but resistant to immunotherapy. Finally, a prognosis model based on three PCDH7-derived genes was constructed (*GPX8*, *BCAR3*, and *TNS4*), which has a good prediction ability on NSCLC patient survival.

PCDH7, known as protocadherin 7, is a subfamily of the cadherin superfamily (Yoshida et al., 1998). PCDH7 has been reported to play a biological role in various cancer types. For instance, Liu et al. discovered that PCDH7 can affect the chemotherapy response of colon cancer, which is regulated by ferroptosis and autophagy (Liu et al., 2022). Wu et al. found that AQP8 could inhibit cancer progression by downregulating PI3K/AKT signaling (Wu et al., 2018). Shishodia et al. found that prostate cancer has a higher level of PCDH7 and could enhance MEK signaling (Shishodia et al., 2019). Wang et al. found that the PCDH7 could be regulated by the circDVL1/miR-412-3p axis and promote renal cancer development (Wang et al., 2022). We found that PCDH7 significantly enhances lung cancer development and is associated with cisplatin resistance, which provides the direction for the potential drug development targeting PCDH7.

We noticed that the EMT pathway was the most enriched biological term in patients with high PCDH7 expression, which indicates the role PCDH7 may exert through EMT mediation. Shen et al. noticed that when EMT activity is reversed, the malignancy and resistance to cisplatin in cisplatin-resistant lung cancer cell lines decrease (Shen et al., 2019). The research result also indirectly highlights the effectiveness of our analysis. Meanwhile, other pathways like apical junction, UV-response, angiogenesis, and TNF-α signaling were also found. These pathways may serve as possible mechanisms mediated by PCDH7 and provide direction for future research. Moreover, PCDH7 was negatively correlated with CD8^+^ T cells and activated NK cells. These two types of cells act as killer cells to suppress cancer in general solid tumors, especially in lung cancer (Guillerey, 2020; Reina-Campos et al., 2021). Therefore, the recruitment of PCDH7 to peripheral cells in the microenvironment of NSCLC may also be the potential mechanism of its role.

With the arrival of the era of biological big data, the rapidly developing bioinformatics has greatly helped relevant researchers (Yu et al., 2021b; Ren et al., 2021). This study identified the potential role of PCDH7 in NSCLC through high-quality data analysis and validated it through further biological experiments. However, some limitations cannot be ignored. First, through specific bioinformatics algorithms, we explored the potential mechanisms by which PCDH7 works through TCGA’s big data. However, bioinformatics results are difficult to truly indicate the actual organizational microenvironment. Therefore, the potential bias generated may reduce the credibility of the conclusion. Second, the vast majority of patients obtained from TCGA are from the Western population. Considering the biological differences between different ethnic groups, the credibility of our results in Asian and African American populations will decrease.

## Data Availability

The original contributions presented in the study are included in the article/Supplementary Materials; further inquiries can be directed to the corresponding author.

## Supplementary material

SUPPLEMENTARY FIGURE S1 Data preprocessing process of GSE21656 and GSE108214.

SUPPLEMENTARY FIGURE S2 Results of qRT-PCR. Notes: **(A)**: RNA level of PCDH7 in normal and lung cancer cell lines; **(B, C)**: knockdown efficiency of PCDH7 in A549 and H1299 cells.

SUPPLEMENTARY FIGURE S3 IHC and subcellular localization of PCDH7. Notes: **(A)**: IHC of PCDH7 in normal lung tissue; **(B)**: IHC of PCDH7 in lung cancer tissue; **(C)**: subcellular localization of PCDH7 (HPA database, U2OS).
