# Supplementary figures and images for: Integrative analysis illustrates the role of PCDH7 in lung cancer development, cisplatin resistance, and immunotherapy resistance: an underlying target
Source: Front Pharmacol. 2023 Jul 19;14:1217213. doi: 10.3389/fphar.2023.1217213 (PMC10394841; doi:10.3389/fphar.2023.1217213)

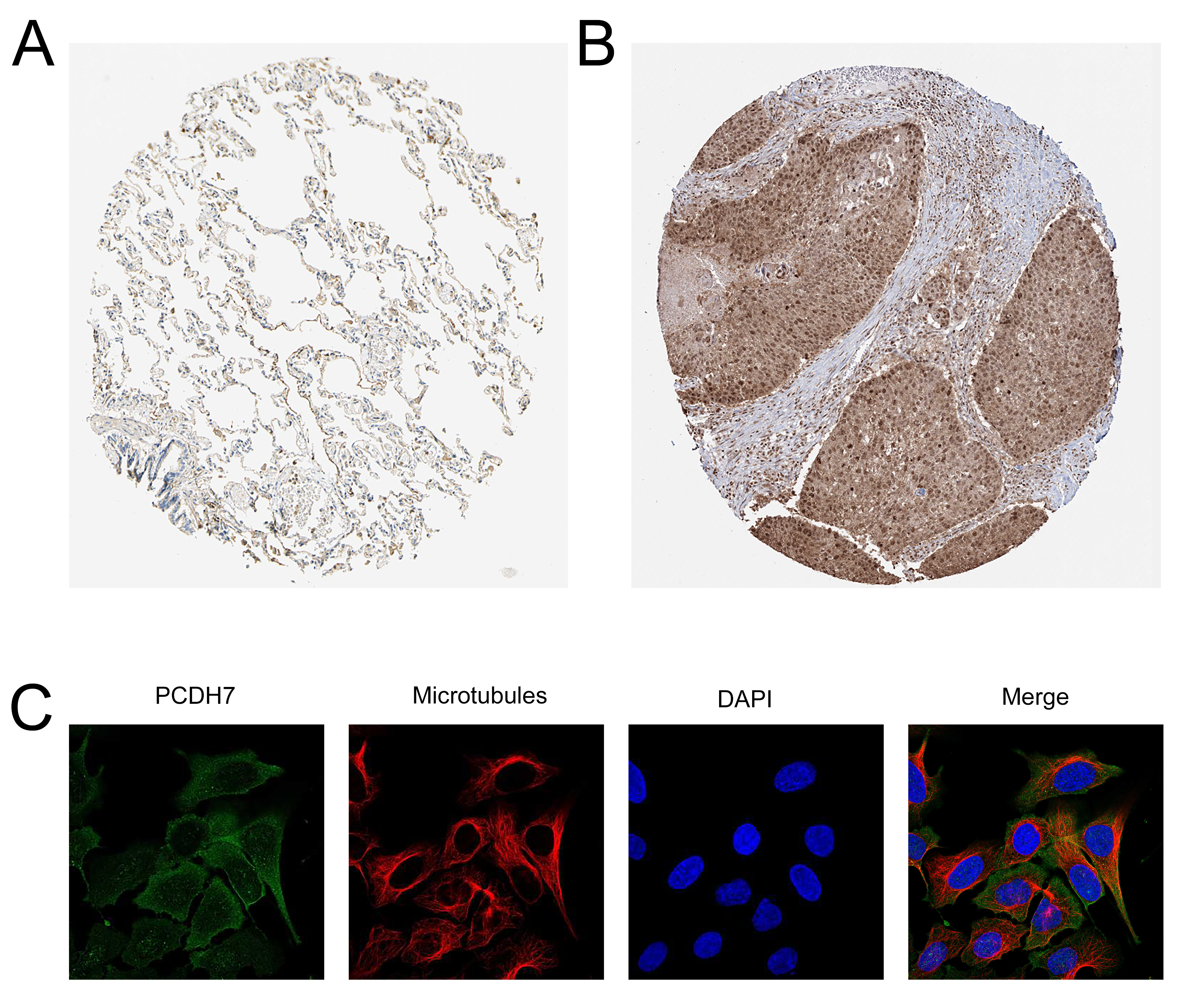

Supplement: Supplementary file 2 [file Image3.TIF]

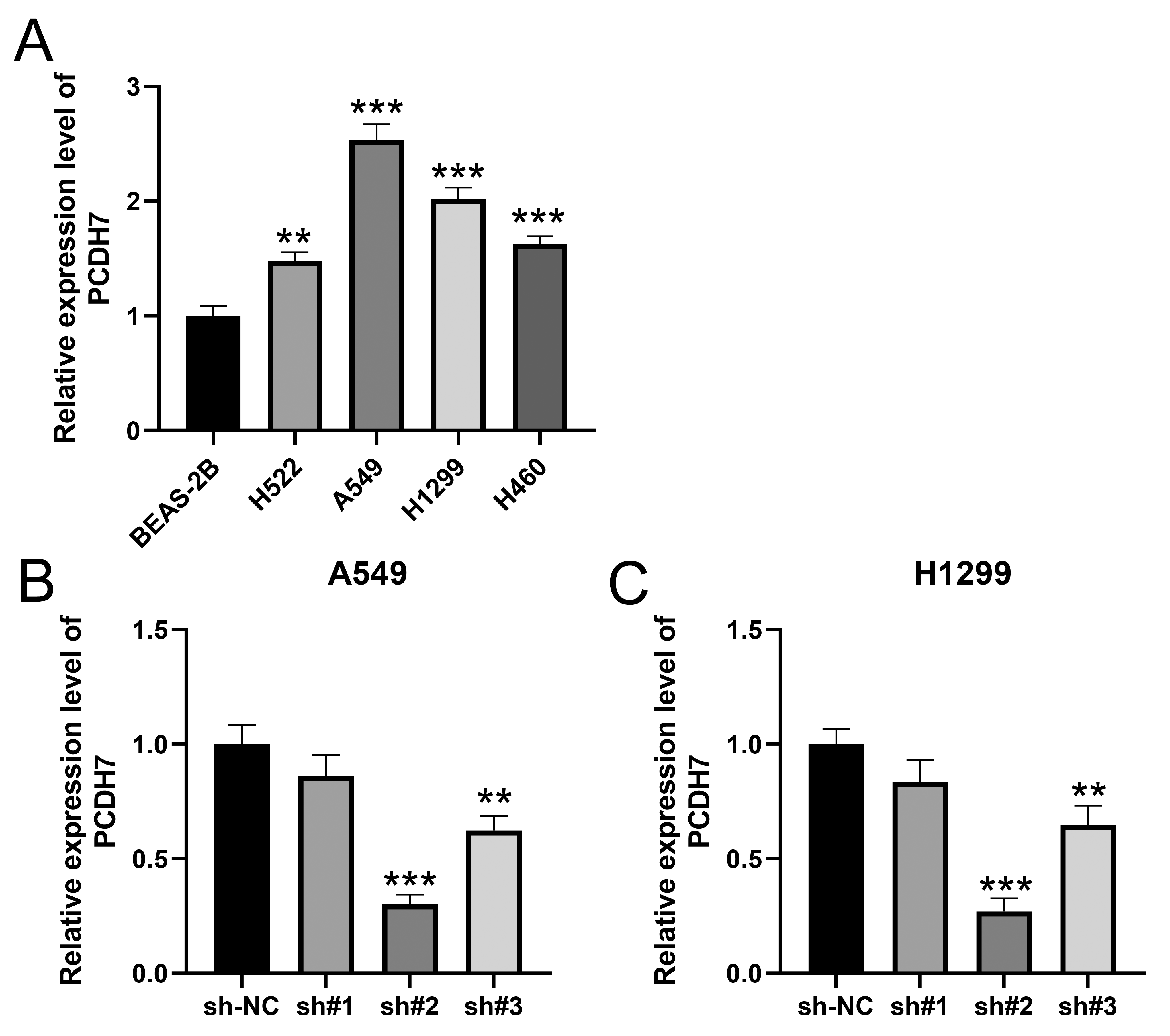

Supplement: Supplementary file 3 [file Image2.TIF]

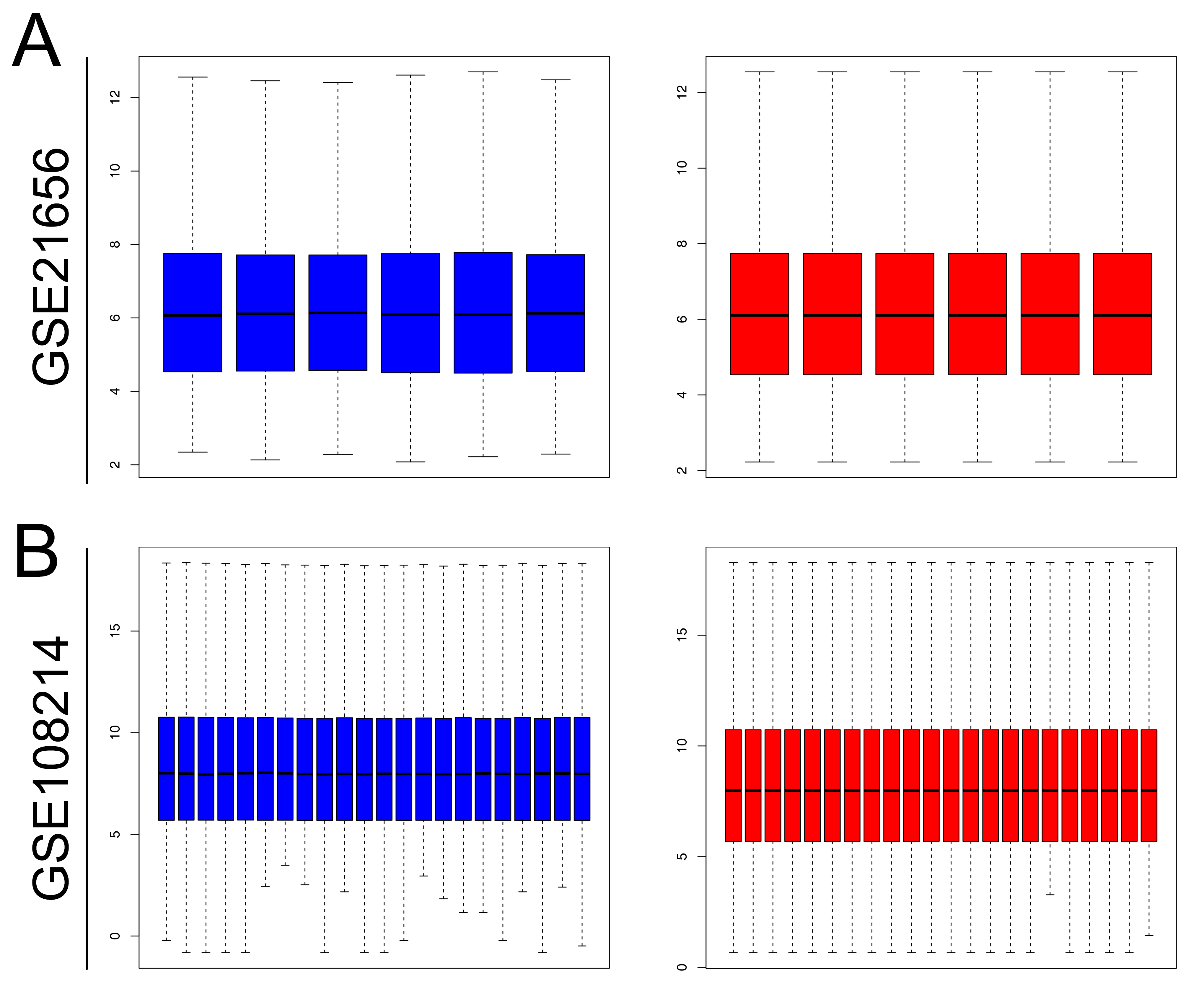

Supplement: Supplementary file 4 [file Image1.TIF]
